# Acute-onset high-morbidity primary photosensitisation in sheep associated with consumption of the Casbah and Mauro cultivars of the pasture legume Biserrula

**DOI:** 10.1186/s12917-017-1318-7

**Published:** 2018-01-11

**Authors:** Jane C. Quinn, Yuchi Chen, Belinda Hackney, Muhammad Shoaib Tufail, Leslie A. Weston, Panayiotis Loukopoulos

**Affiliations:** 1 School of Animal and Veterinary Sciences, Charles Sturt University, Wagga Wagga, NSW 2650 Australia; 2 Graham Centre for Agricultural Innovation; Charles Sturt University and NSW Department of Primary Industries, Wagga Wagga, NSW 2650 Australia

**Keywords:** Photosensitisation, Primary, Unseasonal, *Biserrula pelecinus L.*, Legume, Sheep

## Abstract

**Background:**

Primary photosensitisation (PS) subsequent to ingestion of the pasture legume *Biserrula pelecinus L.* (biserrula) has recently been confirmed in grazing livestock. Given the potential utility of this pasture species in challenging climates, a grazing trial was undertaken to examine if both varieties ‘Casbah’ and ‘Mauro’ were able to cause photosensitisation in livestock, and if this could be mitigated by grazing in winter, or in combination with other common pasture species.

**Results:**

A controlled grazing trial was undertaken in winter in Australia with plots containing a dominant pasture of *Biserrula pelecinus L. cv.* ‘Casbah’ or ‘Mauro’, or mixed biserrula/perennial ryegrass populations. A photosensitisation grading system was established. 167 prime meat ewe lambs were introduced to the plots and monitored twice daily. Mild clinical signs were observed at 72 h on pasture. All animals were removed from biserrula dominant stands at this point. Four animals grazing ‘Casbah’ dominant pasture rapidly proceeded to severe photosensitisation in the following 12 h. Animals remaining on mixed biserrula/ryegrass stands did not exhibit severe PS but showed an 89% incidence of mild to moderate photosensitisation over the following 14 days. Animals on mixed lucerne showed significantly lower PS score than animals grazing biserrula varieties of any composition. The trial was halted at 14 days as only plots with low biserrula proportion still contained unaffected animals.

Necropsy revealed severe multifocal erythematous ulcerations and alopecia of the ear pinnae, severe bilateral periorbital and conjunctival oedema and variably severe subcutaneous facial oedema. No evidence of hepatopathy was present. A diagnosis of acute unseasonal primary photosensitisation caused by biserrula ingestion with no other underlying pathology was confirmed.

**Conclusions:**

We report an unseasonal outbreak of acute photosensitisation in sheep grazing *Biserrula pelecinus L cvs.’*Casbah’ and ‘Mauro’ with exceedingly high morbidity. A grading system is also proposed as a tool for objective and consistent clinical appraisal of future PS outbreaks. This finding expands our definition of seasonal and temporal risk periods for biserrula photosensitisation, and is the first to identify that both commercial cultivars of biserrula can cause primary photosensitisation in sheep.

## Background

*Biserrula pelecinus* is a self-regenerating annual legume highly suited to low to medium rainfall zones [16]. It is able to adapt to a broad range of soil types with higher productivity compared to other legumes [20]. *Biserrula pelecinus* L. var. ‘Casbah’ was commercialised in 1997, and has been widely used by farmers in Australian mixed farming systems as a break crop, especially in South Australia and New South Wales [14, 23–26]. Anecdotal reports of cases of photosensitisation in livestock associated with biserrula ingestion had been noticed since this legume was commercialised in 1997 as a highly productive pasture with multiple agronomic advantages [17]. However, it was only in the recent past that a direct connection between exposure to biserrula pasture and onset of primary photosensitisation was confirmed by detailed diagnostics [4].

Photosensitisation (PS) is a skin disorder caused by photodynamic pigments activated by long-wavelength ultraviolet or visible light in exposed areas of skin, resulting in dermatitis [1]. In livestock, areas commonly affected include those lacking protective fleece, hair coat or skin pigmentation such as the muzzle, face, ears, eyes, mammary glands and genitalia [1].

In this study, we present an outbreak with exceedingly high morbidity in sheep grazing the Mediterranean pasture legume *Biserrula pelecinus L.* during its vegetative stage, in which animals exhibited an unexpected acute primary photosensitisation during mid-winter. Dissimilar to the current report, all but one [4] of the previous reports of PS outbreaks were reported in spring, a season classically associated with presentation of photosensitivity in livestock. Winter is not traditionally a time when photosensitisation outbreaks present to the livestock veterinarian. There are a number of reasons why this is the case: 1) lack of significant UV exposure due to cloudy conditions and short day length; 2) lack of actively growing plants, the phase during which toxic secondary compounds are commonly generated; 3) increased skin cover by hair or fleece for seasonal protection against colder conditions.

Furthermore, the clinical course and the speed of onset of clinical photosensitisation subsequent to exposure or ingestion of the presumptive or established aetiologic agent has not been clearly defined or evaluated. This is the first report of primary photosensitisation in sheep resulting from ingestion of both commercially available varieties of Biserrula, namely ‘Casbah’ and ‘Mauro’, in mid-winter, the presentation of which was acute, severe and with exceedingly high morbidity. We also define a clinical grading scale that is capable of differentiating mild, moderate and severe PS as a tool for objective and consistent clinical appraisal of PS outbreaks in the future, and describe the clinical course in relation to the severity of PS and the causative agent. This report also identifies that mixed pastures are not a guaranteed protection against incidence of PS in animals grazing this pasture species. This report highlights the need to consider photosensitisation as a differential diagnosis, even during winter months, when cases of acute dermatitis are rarely reported in grazing livestock.

## Methods

### Pastures

**Fig. 1:**
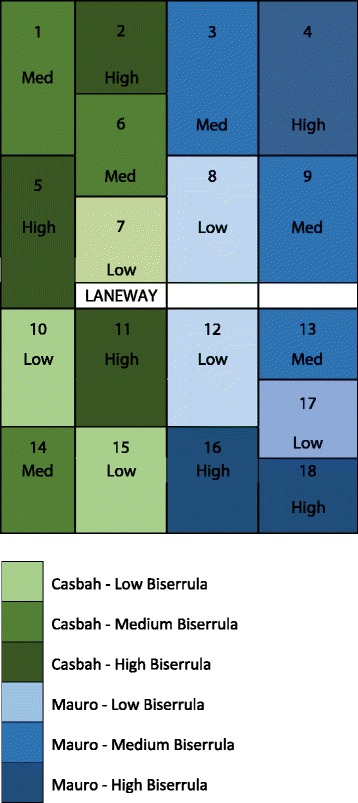
Randomised block design of regenerating biserrula pasture plots used for the winter biserrula grazing trial. Two varieties of biserrula were sown in the preceding year: ‘Mauro’ and ‘Casbah’. Plots were established in triplicate with a total of 9 plots per variety. Blue: ‘Casbah’; green: ‘Mauro’. Low Biserrula: Biserrula pastures oversown with annual ryegrass (*Lolium rigidum*) at a rate of 40 kg/ha. Medium Biserrula: Biserrula pastures oversown with annual ryegrass at a rate of 120 kg/ha. High Biserrula: Biserrula pastures containing no or very low contamination with other pasture species (<10%) and were considered biserrula-dominant

Two varieties of *Biserrula pelecinus* L.*, cv.* ‘Casbah’ and ‘Mauro’, were used under commercial conditions in an established long-term cropping paddock (−35.03°, 147.34°) to be available for 2015 spring grazing. The study site was owned and managed by the Charles Sturt University Farm, and no additional permission was required for experimentation on this site. Planting occurred in a paddock that was previously sown with dual purpose wheat in as per usual commercial practice. The site contained up to 10% infestation of annual ryegrass (*Lolium rigidum*) that exhibited resistance to multiple classes of post-emergent herbicides. A very low infestation of common pasture weeds was also present (<5%). All trial plots were sown according to standard commercial practices on 16 May 2015. Prior to sowing, plots were treated with a pre-emergent herbicide and then the stubble burned and light tillage performed. Trial sites were sown with 10 kg/ha of commercially available scarified biserrula seed of the two biserrula varieties ‘Casbah’ and ‘Mauro’ with 8 kg/ha ‘biserrula special’ inoculant (Alosca Technologies, Australia) for rhizobial establishment. Plots were treated post-sowing with Talstar insecticide (bifenthrin) at 100 ml/ha on 21 May 2015 to prevent insect herbivory post emergence. In order to establish pastures containing mixed populations of biserrula and ryegrass as a mitigating fodder, plots were oversown with annual ryegrass (*Lolium rigidum*) at a rate of 120 kg/ha (“medium biserrula” plots) or 40 kg/ha (“low biserrula”). Control plots (“high biserrula”) contained no or very low contamination with other pasture species (<10%) and were considered biserrula-dominant. All plots were over-sown in triplicate using a random mixed block design. Plots ranges in size from 0.21 ha (plot 2, 6 & 7) to 0.38 ha in size (plots 1, 13, 17, 18). The plot design and pasture composition is shown in Fig. 1.

### Animals

One hundred sixty seven ‘Primeline’ meat ewe lambs were sourced at 10 months old from a local producer. Prior to purchase for this study, animals were maintained according to standard industry practices on a local property and were grazing mixed annual ryegrass pasture. Inclusion criteria for the trial included a healthy appearance at yarding with no visible pathology. All animals were naive for exposure to biserrula pasture. Prior to entry, animals were yarded and weighed (mean entry weight, 35.1 kg). All lambs were drenched prior to entry to pasture according to manufacturers recommendations (Ivomec®, Merial Australia). Water was provided ad libitum via automatic drinkers to all plots. As a control, an established mixed lucerne *(Medicago sativa)*/perennial ryegrass *(Lolium perenne)*/subclover (*Trifolium subterraneum*) paddock adjacent to the sown biserrula trial site was used as an industry-standard comparison pasture.

### Photosensitisation grading system

To quantify clinical signs and severity of photosensitisation a de novo photosensitisation clinical scoring and grading system was designed (Table 1). The presence and severity of PS associated lesions in the face, eyes, ears and body were evaluated separately for each of these regions and scored o a scale of 0 to 5, as shown in Table 1. The four individual scores were then added to produce a PS grade. PS was defined as being mild, moderate or severe if having a composite PS score of <7, <12 or ≥12 respectively.

**Table 1:** Proposed photosensitisation clinical grading system. Lesions were defined as being mild, moderate or severe if having a composite score of <7, <12 or ≥12 respectively

Score	Lesion description			
	Face and muzzle	Eyes	Ears	Fleece/body
0	No apparent lesions	No apparent lesions	No apparent lesions	No apparent lesions
1	Mild cutaneous oedema and erythema	Mild serous blepharitis	Drooping of ears with mild oedema.	Mild erythema of exposed areas.
2	Cutaneous oedema and erythema; mild to mderate aural and facial oedema	Serous exudation, mild to moderate perorbital oedema and conjunctival erythema	Mild aural pitting oedema	Marked erythema of exposed areas.
3	Severe cutaneous erythema, crusting and black discolouration; moderate aural and facial oedema	Serous exudation, possible crusting; marked palpebral and conjunctival erythema and oedema; mild to moderate periorbital oedema	Marked aural pitting oedema, curling of ear ends, some flaking or other lesions possible	Multifocal, possibly multifocally extensive, patchy fleece loss, erythema of underlying skin.
4	Severe cutaneous erythema, crusting and black discolouration; severe aural and facial oedema	Severe perorbital, palpebral and conjunctival oedema. Possible corneal opacity or ulceration or opacity, serous exudate, eyelid crusting	Marked aural pitting oedema, curling of ear ends, other dermal lesions present. Skin flaking and multifocal necrosis; some tissue loss from rubbing may be evident; moderate serous exudation	Marked focal or multifocal fleece loss and dermatitis of exposed skin.
5	Multifocal irregularly shaped cutaneous necrosis and exudation; severe dermatitis including secondary lesions; severe facial oedema	Severe periorbital, palpebral and conjunctival oedema; eyes closed; corneal ulceration and/or opacity	Marked aural pitting oedema, curling of ear ends and tissue loss, other dermal lesions present including flaking and multifocal necrosis; abundant serous exudation	Widespread fleece loss, severe dermatitis of exposed skin. Significant fleece loss in dorsum and flanks.

### Grazing trial

A total of 167 lambs was randomly assigned to pasture in July (mid-winter) 2015 at matched stocking densities using restricted randomisation by weight (6–7 animals per plot; approximately 14 DSE/ha) (Fig. 1), 53 grazing in plots containing the biserrula variety ‘Mauro’ and 65 grazing on the variety ‘Casbah’. A further 49 lambs were grazing the non-PS mixed lucerne pasture. The ‘*Mauro*’ dominant pasture contained 88% biserrula by composition (*n* = 18 animals); while the biserrula ‘*Casbah*’ dominant pasture contained 81% biserrula by composition (*n* = 21 animals). Medium biserrula pastures of both varieties contained between 52 and 75% biserrula by composition (‘Mauro’ medium, n = 18 animals; ‘Casbah’ medium, *n* = 23 animals). Low biserrula pastures contained <50% biserrula species (‘Mauro’ low, *n* = 17 animals; ‘Casbah’ low, n = 21 animals). Animals were monitored twice daily for behavioural or physical changes (shade-seeking behaviour, reduction in grazing or general activity, physical isolation, lameness) and scored every 3 days for clinical signs of photosensitisation.

All lambs were introduced to pastures on 9 July 2015 with all remaining animals removed from trial plots on 23 July 2015. Animals exhibiting clinical signs of photosensitisation were removed from the trial on the date of observation and scored for clinical signs of PS. Withdrawn animals were removed to a non-photosensitising pasture (lucerne/ryegrass/subclover) with full availability of shade and water. Once less than 50% of the original pen cohort were remaining in any plot, all members of that cohort were removed from the trial. Any animal with a PS score > 11 was selected for euthanasia. All animals were carefully monitored both within and after the trial for increasing signs of PS. PS was observed to resolve as soon as animals were withdrawn from biserrula pastures. No other clinical intervention was required. This trial was approved and compliant with requirements of the Charles Sturt Institutional Animal Care and Ethics Committee (Protocol 13/018).

### Statistical analysis

Photosensitisation scores were compared between cohorts using ANOVA and general mixed linear models using SPSS™ (IBM, Version 20), with variety, plot, composition and time as fixed factors. Significance was defined as *p* < 0.05.

## Results

### Clinical course of the photosensitisation outbreak

Initially, animals grazing biserrula ‘Casbah’ dominant pastures were observed to exhibit some reduced grazing behaviour compared to other plots, showed less apparent activity within the plots and some individuals exhibited overt shade-seeking behaviour such as standing in a close line, nose to tail, or lying close to troughs or fence poles.

The first clinical signs of photosensitisation, ranging from mild to severe, were observed in 5 animals after only 3 days on biserulla dominant pasture. Three animals were located in the plot containing the highest contribution of biserrula ‘*Casbah*’ (plot 4, 98% composition, PS scores 2.5, 2.5 and 15), one was in the second highest contribution ‘*Casbah*’ plot (8, 92% composition, PS score 2.5), and one in a dominant ‘*Mauro*’ plot (plot 7, 90% composition, PS score 4). All five lambs exhibited swelling and drooping of the pinnae and blepharitis and showed shade-seeking behaviour. No PS was observed in any animal on mixed lucerne pasture at this timepoint.

**Fig. 2:**
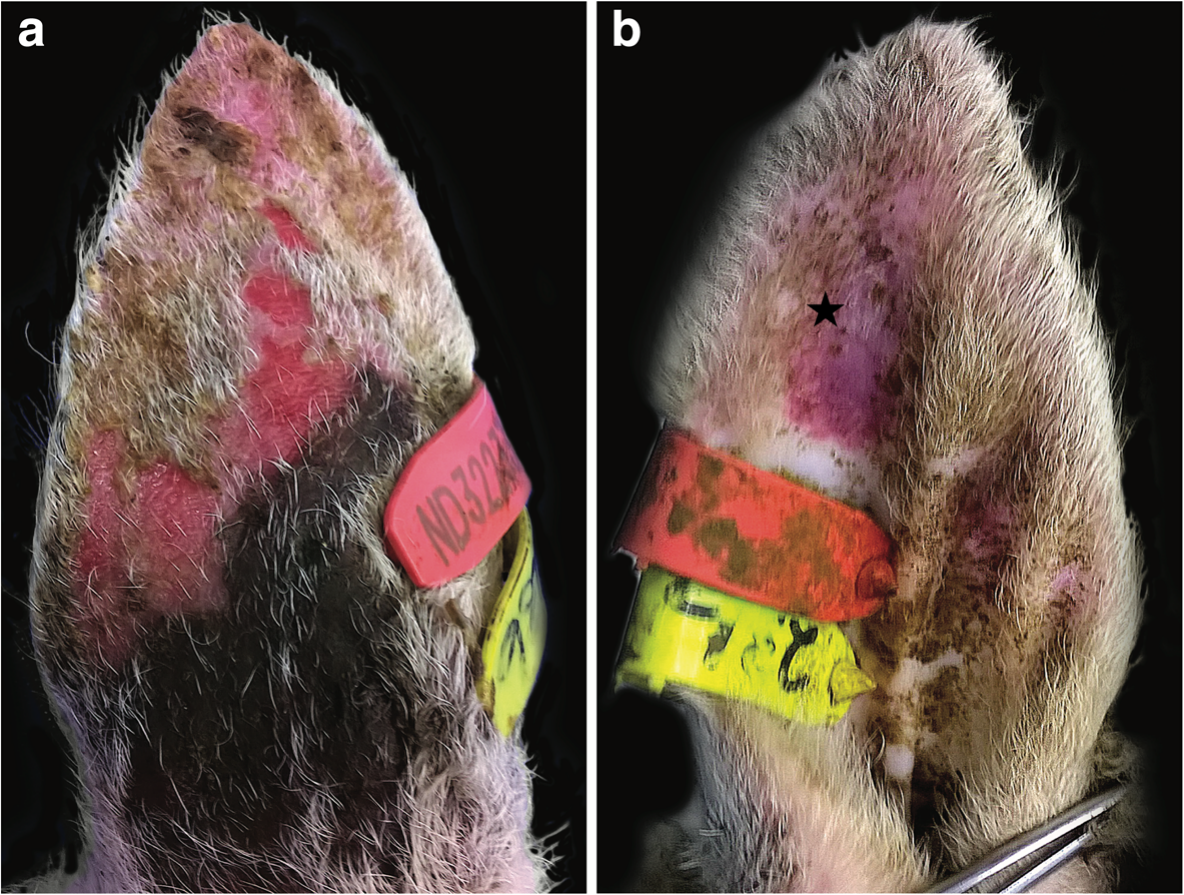
Skin lesions associated with primary photosensitisation caused by ingestion of biserrula. **a** External aspect of left ear pinna: multifocal to coalescing erosion, ulceration and erythema; (**b**) Inner surface of the tip of the ear: erythema and alopecia (star). Skin covered by ear tags was not affected

All those animals identified to be showing clinical signs of photosensitisation on day 3 of the grazing period were removed from the trial. Despite removal, two of the animals (animals 1 and 2) showed clinical signs which either remained severe (PS score 10.5) or increased in severity (PS score 4 increasing to 7). The most severely affected animal (animal 1) (Plot 4, high Casbah plot, PS score 15) was selected for necropsy, while animal 2 was closely observed and was observed to resolve over the following 7 days. Whilst oedema of the pinnae and muzzle and blepharitis were observed in other cases, extensive facial oedema was only apparent in animal 1 (Fig. 2). Venous blood was collected for haematology and biochemistry, faecal samples for analysis of parasite burden, and the animal was submitted for full diagnostic work up.

Clinical photosensitisation (mild, moderate or severe) was observed in 100% (*n* = 39) of animals grazing either ‘*Mauro’* or ‘*Casbah’* dominant plots by day 6, facilitating complete withdrawal of all animals on biserrula dominant pastures at this time. Comparison between biserrula dominant pastures at day 6 showed that the animals on ‘Casbah’ had significantly higher PS score compared to the animals on ‘Mauro’ at this time point (*p* = <0.01). No animals showed signs of icterus.

In total, 107/118 (91%) animals on biserrula pastures showed some clinical signs of photosensitisation during the 14 day monitoring period. On day 14, the majority of animals showed composite PS scores ranging from 0 to 7.5 with most within the 0–5 range, indicating mild photosensitisation was apparent in the majority of animals that had remained on the plots until day 14 (*n* = 68; mean PS score 2.4). Only four moderately affected were identified on day 14 of the grazing trial, all were present in plots containing a medium composition of either *B. pelecinus* L. ‘Casbah’ or ‘Mauro’ (Plot 3, Casbah medium: PS scores 9 and 10; Mauro medium: plot 5, PS scores 9 and 11).

### Effect of pasture and biserulla composition and biserulla variety on photosensitisation severity

Mean PS score for animals grazing mixed lucerne pastures at day 14 was 0 (zero) with animals on these pastures showing significantly lower PS score than animals grazing biserulla varieties of any composition (*p* ≥ 0.0001). Exit weight was not found to be statistically significant between the different pastures by variety or composition.

At day 14, biserulla variety was found to have a significant effect on severity of PS, with ‘Casbah’ showing significantly higher PS values than ‘Mauro’ (‘Casbah’ mean PS score: 3.09 ± 0.30, *n* = 46; ‘Mauro’ mean PS score: 1.97 ± 0.35, *n* = 34, F = 5.776; *p* = 0.019). Composition was also shown to exert a significant effect, with the medium biserulla composition mean PS score being significantly higher than the low biserulla low composition mean PS score (2.89 ± 0.34 vs: 2.31 ± 0.33, F = 6.332, *p* = 0.001).

### Clinical pathology findings

Complete blood count of animal 1 was consistent with the presence of a mild inflammatory process, showing mild leucocytosis due to neutrophilia and monocytosis (white blood cell count 17.4 × 10^9^/L, reference range 4.1–13.0 × 10^9^/L; neutrophils 11.5 × 10^9^/L, reference range 0.5–9.3 × 10^9^/L; monocytes 1.1 × 10^9^/L, reference range 0.0–0.7 × 10^9^/L), likely attributable to the aural dermatitis. Serum biochemistry showed mildly elevated AST (157 U/L, reference range 87–156 U/L) and CK (600 U/L, reference range 91–472 U/L), without elevation of GGT (52 U/L, reference range 35–61 U/L) or bilirubin (2 μmol/L, reference range 4–15 μmol/L). These changes were unremarkable. Faecal egg count was negative.

### Gross pathology findings

**Fig. 3:**
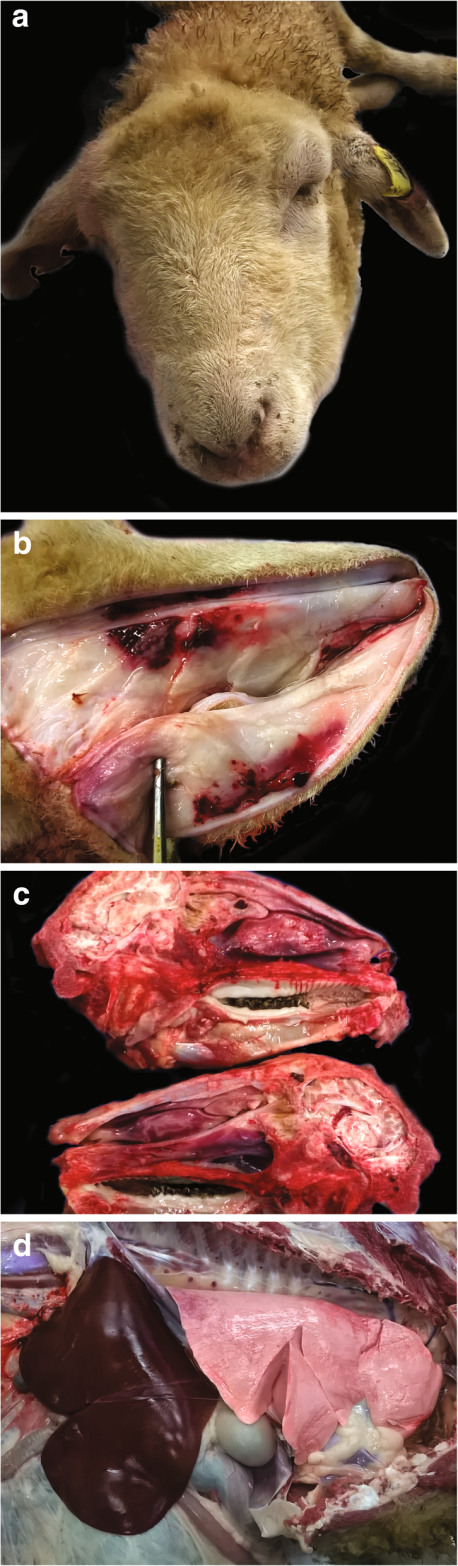
**a** Severe bilateral periorbital and conjuctival oedema and variably severe subcutaneous facial oedema. **b** Severe focally extensive haemorrhage in the nasal subcutaneous tissues. **c** Severe narrowing of the nasal cavity due to oedema. **d** No significant changes were observed in the liver and other internal organs

Necropsy of animal 1 revealed multifocal to coalescing erythematous ulcerations, hair loss and crusting on the external aspect of both ear pinnae. The areas of ear skin which had been protected from UV exposure by the animal’s identification tag were unaffected (Fig. 2a, b). Moderate erythematous and alopecic patches were present on the internal aspect of the left ear pinna. Severe bilateral periorbital and conjunctival oedema and variably severe subcutaneous facial oedema were noted, the latter ranging from moderate to severe in the occipital region, severe in the nasal region, to exceedingly severe in the mandibular region (Fig. 3a). In the nasal subcutaneous tissues, there was severe focally extensive haemorrhage (Fig. 3b). Severe blepharitis was observed bilaterally but no gross corneal damage was observed. The nasal mucosa was extensively congested and the submucosa was oedematous and congested, both changes sparing the ethmoid area (Fig. 3c). No significant changes were observed in other organs, including the liver (Fig. 3d).

### Histopathology findings

**Fig. 4:**
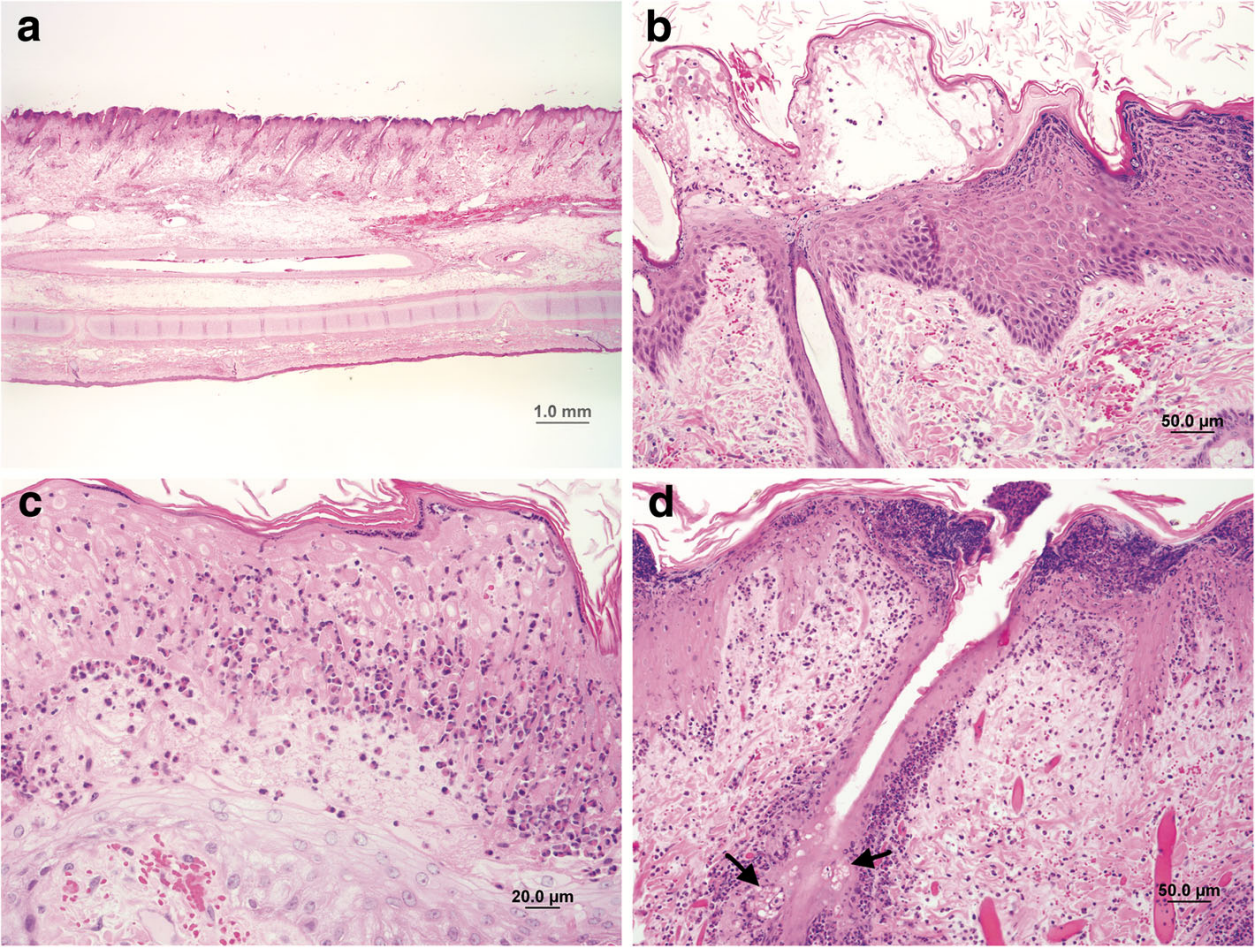
Photomicrographs of the alopecic and oedematous areas of the ear pinnae: (**a**) Low magnification showing severe haemorrhage and oedema in the dermis. H&E, objective × 1.25. **b** Micropustules containing neutrophils, eosinophils and necrotic debris in the stratum corneum and upper stratum granulosum on the external aspect of the ear pinna. Mild individual cell keratinocyte necrosis, mild acanthosis and haemorrhage in the upper dermis are also shown. H&E, × 200. **c** Moderate zonal necrosis of the outer layers of the epidermis, which is infiltrated by numerous eosinophils and neutrophils, at places forming small intraepidermal pustules. Keratinocytes show moderate individual cell necrosis. H&E, × 400. (**d**) Severe epidermal necrosis with obliteration of the follicular epithelial structure and sebaceous gland necrosis (arrows) in the most severely affected section of the ear pinna. Intraepidermal nodular pustules formed by dead and viable neutrophils; eosinophils can be seen multifocally. The dermis is infiltrated by eosinophils and neutrophils, particularly adjacent to necrotic hair follicles. H&E, × 200

Histopathological examination of ear lesions from animal 1 revealed similar changes. These included exceedingly severe neutrophilic and eosinophilic epidermitis; dermatitis with intradermal nodular pustules containing neutrophils, eosinophils, macrophages and necrotic keratinocytes; severe haemorrhage and oedema; and individual keratinocyte to transmural epidermal necrosis. In the most severe lesions, moderate multifocal coagulative necrosis of sebaceous glands was noted (Fig. 4). The liver showed minimal periportal and occasionally periacinar lymphoplasmacytic aggregation. The heart, lungs, kidneys and brain showed no noteworthy changes.

## Discussion

Previous reports of photosensitisation in lambs grazing biserrula identified dermal lesions of the face and ears [4, 15, 18] suggesting established or resolving lesions. Kessell et al. (2015) [4] also presented a PS outbreak due to biserrula Casbah in winter, affecting 25% of animals. However, the high morbidity, the speed of onset (4 days on pasture) and severity of presentation of PS described in our study have not been reported for this pasture species previously [2, 4, 18–20]. This study is the first to define the speed of onset of clinical photosensitisation subsequent to animals ingesting biserrula (<72 h). This study also identifies a change in behaviour in otherwise subclinically affected animals that could be used to predict the onset of clinical cases. Together these findings identify unique aspects of this clinical entity.

Prior to this experiment, it was not known whether both varieties of biserrula commercially available in Australia, ‘Casbah’ and ‘Mauro’, were able to induce photosensitisation. Previous reports had identified the variety ‘Casbah’ to be phytophototoxic, but it was not known if ‘Mauro’ was also able to exert this effect. The current report identified that both varieties can induce photosensitisation in sheep grazing on them with the vast majority of animals showing clinical signs on either variety at both low and high composition densities. Such high morbidity rates have also not been reported previously nor has the severity of clinical presentation been evaluated systematically in prior outbreaks [4, 13–16]. Together our data suggest that a low composition pasture of *B. pelecinus L* cv. ‘Mauro’ might be the least photosensitising option available to producers, although neither variety is inert in its effect at a composition above 25% of total pasture.

Clinical photosensitisation occurs in three forms. Type I (primary PS) results from the direct ingestion, or exposure by dermal contact to, photodynamic compounds found within certain plant species, including biserulla, *Froelichia humboldtiana*, and alfalfa [2–5]. Type II, not reported in sheep to date [6], is associated with congenitally abnormal porphyrin metabolism. Type III (hepatogenous) is the most common form of PS in livestock, and is caused by impaired liver function resulting in failure to excrete circulating phylloerythrin, a natural breakdown product of chlorophyll [7–12], which in turn causes phototoxic damage to the dermal and subdermal layers of exposed skin.

This study, and previous case studies and reports on biserulla, showed no evidence of hepatopathy [4, 13–16]. It is therefore suggested that biserrula cultivars contain photodynamic agents that lead to the onset of primary photosensitisation, occurring when the plant reaches show reproductive maturity [2, 4, 13, 15, 18, 21]. Although photoactive metabolites involved in this syndrome have not all been fully structurally elucidated, bioassay-guided isolation and analysis by NMR and UV spectroscopy along with mass spectrometry have revealed structural features of at least three bioactive metabolites present in the shoot extracts of both biserrula cultivars (LW, JQ unpublished data) and in seasonal equivalence to spring – early summer in NSW Australia. Photodynamic molecules such as these are polycyclic ring structures with conjugated bonds, generating potential electronic cycling and free radicals following exposure to UV irradiation, thereby leading to photosensitisation [2, 4]. At this time, investigation and structural elucidation is underway to 1) identify the photodynamic constituent(s) present in fresh *B. pelecinus* L. foliage and foliar extracts and 2) to investigate the potential to screen and select for less photocytotoxic genotypes for use in Mediterranean climates, including Australia.

The current study identifies the earliest phenological presentation of clinical photosensitisation to occur well prior to the reproductive phase of the plant’s life cycle, indicating that the currently unidentified photodynamic compound that causes photosensitisation may exist across all phenological stages of biserrula growth.

A diagnosis of photosensitisation is based on history, clinical signs and the exclusion of all other possible dermatopathies [1, 22]. Furthermore, the acute onset of symptoms in winter, when extensive cloud cover was present, in conjunction with the consumption of a known primary photosensitising plant species, largely rules out sunburn as a differential diagnosis in this case.

In this case, the eosinophilic infiltration of the epidermis and dermis could be a tissue response to necrotic keratinocytes, but it could also be a reaction to the deposited photodynamic substances in the skin, either through ingestion or possibly also by dermal contact in affected animals. Eosinophilic inflammation has not been reported as a histopathological finding in primary photosensitisation previously [1], and may represent an undetermined component involved in the aetiopathogenesis. The high morbidity rate of primary photosensitisation observed in the feeding trial (91% overall), combined with the pathologic findings in this case are highly suggestive of the presence of photosensitising compound(s) in sufficient quantity to cause severe dermatitis even in seasons of low ambient temperatures and lower UV exposure, conditions which are not usually associated with outbreaks of photosensitisation in livestock.

## Conclusion

The high incidence of primary photosensitisation combined with the pathologic findings strongly suggest the presence of photosensitising agent(s) in sufficient quantity in both biserrula cultivars. Severe dermatitis in grazing sheep was observed unexpectedly during late winter growth conditions in central NSW, when low ambient air and soil temperatures and variable UV exposure were experienced.

Finally, the design and introduction of a quantitative grading system, such as the one proposed and applied in this report, to evaluate the severity of photosensitisation lesions will facilitate the objective evaluation, monitoring and comparison of photosensitisation cases in the future.

## Abbreviations

AST: Aspartate aminotransferase
CK: Creatine kinase
DSE: Dry sheep equivalent
EDTA: Ethylenediaminetetraacetic acid
GGT: Gamma-glutamyl transferase
H&E: Haematoxylin and eosin
ha: Hectare
PS: photosensitisation
